# Identification of Depression Subtypes in Parkinson's Disease Patients via Structural MRI Whole‐Brain Radiomics: An Unsupervised Machine Learning Study

**DOI:** 10.1111/cns.70182

**Published:** 2025-02-06

**Authors:** Zihan Zhang, Jiaxuan Peng, Qiaowei Song, Yuyun Xu, Yuguo Wei, Zhenyu Shu

**Affiliations:** ^1^ Jinzhou Medical University Postgraduate Education Base (Zhejiang Provincial People's Hospital, People's Hospital of Hangzhou Medical College) Hangzhou Zhejiang China; ^2^ Center for Rehabilitation Medicine, Department of Radiology Zhejiang Provincial People's Hospital, People's Hospital of Hangzhou Medical College Hangzhou Zhejiang China; ^3^ Advanced Analytics, GE Healthcare Hangzhou China

**Keywords:** cluster analysis, depression, magnetic resonance imaging, Parkinson's disease, radiomics

## Abstract

**Objective:**

Current clinical evaluation may tend to lack precision in detecting depression in Parkinson's disease (DPD). Radiomics features have gradually shown potential as auxiliary diagnostic tools in identifying and distinguishing different subtypes of Parkinson's disease (PD), and a radiomic approach that combines unsupervised machine learning has the potential to identify DPD.

**Methods:**

Analyze the clinical and imaging data of 272 Parkinson's disease (PD) patients from the PPMI dataset, along with 45 PD patients from the NACC dataset. Extract radiomic features from T1‐weighted MRI images and employ principal component analysis (PCA) for dimensionality reduction. Subsequently, apply four unsupervised clustering methods including Gaussian mixture model (GMM), hierarchical clustering, K‐means, and partitioning around medoids (PAM) to classify cases in the PPMI dataset into distinct subtypes. Identify high‐risk subtypes of DPD on the basis of the time and number of depression progression, and validate these findings using the NACC dataset. The data from the high‐risk subtype were divided into a training subtype and a testing subtype in a 7:3 ratio. Multiple logistic regression analysis was conducted on the training subtype data to develop a traditional logistic regression model for the high‐risk subtype, which was subsequently compared with a supervised logistic regression model constructed for the entire PPMI cohort. Finally, the performance of both models was evaluated using receiver operating characteristic (ROC) curves. In addition, a decision tree (DT) model was constructed based on independent risk factors of high‐risk subtypes and validated using low‐risk subtype data. ROC curves were employed to validate this model across training subtype, testing subtype, and low‐risk subtype datasets.

**Results:**

The PAM clustering method demonstrates superior performance compared to the other three clustering methods when the number of clusters is 2. High‐risk subtypes of DPD can be effectively distinguished in both the PPMI and NACC datasets. A traditional logistic regression model was developed based on rapid‐eye‐movement behavior disorder, UPDRS I score, UPDRS II score, and ptau in high‐risk subgroups. This model exhibits a diagnostic efficacy (AUC = 0.731) that surpasses that of the traditional regression model constructed using the entire PPMI cohort (AUC = 0.674). The prediction model based on high‐risk subtypes had AUC values of 0.853 and 0.81 in the training and testing subtypes, sensitivities of 0.765 and 0.786, and specificities of 0.771 and 0.815, respectively. The AUC, sensitivity, and specificity in the nonhigh‐risk subtype were 0.859, 0.654, and 0.852, respectively.

**Conclusion:**

By identifying MRI structural radiomics and clinical features as potential biomarkers, the radiomic approach and UCA provide new insights into the pathophysiology of DPD to support the clinical diagnosis with high prediction accuracy.

## Graphical abstract

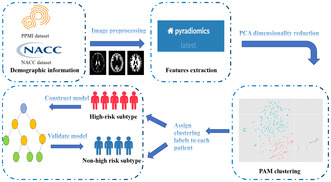

## 1 Introduction

Depression is a common psychiatric symptom of Parkinson's disease (PD) and one of the earliest prodromal complications. Depression not only significantly affects the quality of life of PD patients but also may accelerate the progression of the disease. Owing to the overlapping clinical symptoms of these two diseases, identifying depression in Parkinson's disease (DPD) patients remains challenging at present [1]. On the other hand, the efficacy of medication and psychotherapy in treating DPD is still limited [2]. Therefore, timely identification and classification of DPD subtypes for collaborative management are crucial for better understanding of the disease mechanisms and developing personalized treatment plans.

Magnetic resonance imaging (MRI) is an important noninvasive method for studying changes in brain structure and function, especially structural magnetic resonance imaging (sMRI), which can provide high‐resolution information on brain tissue structure and capture subtle structural changes in the brain. This provides rich information resources for studying PD and its related symptoms [3]. However, in early PD patients, the structural changes in the brain are very subtle, and the characteristic changes in sMRI images cannot be observed with the naked eye. Only in the late stage of the disease can a significant reduction in local brain tissue volume be observed [4]. Although some functional MRI sequences, such as neuromelanin‐sensitive MRI, quantitative magnetization mapping (QSM), resting‐state functional MRI (rs‐fMRI), and diffusion tensor imaging (DTI), can capture early brain changes in PD patients, the application of these techniques is highly dependent on changes in scanners and collection protocols [5].

In recent years, radiomics has gradually been applied as an emerging research field in the study of neurological diseases [6]. Radiomics can extract many quantitative features from image data combined with machine learning (ML) methods to explore image information in detail and reveal potential biomarkers of diseases [7]. Our previous studies also used whole‐brain imaging with sMRI to predict the disease progression of PD and reported that differences in white matter microstructural changes between mild and moderate PD patients were correlated with cognitive function decline [8]. The above research further proves the application value of radiomic analysis in the field of PD research. Although some studies have attempted to use radiomics to distinguish DPD patients [9], there is still limited research on identifying DPD subtypes. On the other hand, most radiomic studies adopted supervised ML methods, which require data to be labeled in advance, and the acquisition of high‐quality labeled data in clinical practice often faces many challenges, especially in multicenter and large‐scale studies [10]. Therefore, exploring the application of unsupervised ML (UML) methods in DPD subtype recognition has important theoretical and practical significance. UML methods do not rely on prelabeled data labels but rather classify and cluster data by analyzing their inherent structure and patterns. This method can not only reveal potential patterns in the data but also reveal the heterogeneity of diseases, which has unique advantages for subtype identification of complex diseases [11]. On the basis of the above theoretical foundation, we assumed that the radiomic features obtained from conventional T1WI magnetic resonance imaging can also identify and classify high‐risk subtypes of DPD through unsupervised cluster analysis and can predict the high‐risk population of PD patients who may progress to DPD through relevant clinical feature analysis.

The main objective of this study was to identify high‐risk subtypes of DPD caused by UML via radiomic data from magnetic resonance structural images. Additionally, a predictive model was constructed on the basis of high‐risk subtypes to identify high‐risk patients with PD who may progress to DPD. Through this study, we hope to reveal the heterogeneity of DPD patients and identify subtypes of depression with different clinical and biological characteristics, thereby providing new ideas for the early diagnosis and personalized treatment of DPD.

## 2 Materials and Methods

### 2.1 Demographic Information

The case data included in this study came from the Parkinson's Progression Markers Initiative (PPMI) (http://www.PPMI‐info.org) and the National Alzheimer's Coordinating Center (NACC) (https://naccdata.org) dataset. The PPMI is the first global collaboration of researchers, funders, and research participants dedicated to identifying biomarkers to improve Parkinson's disease treatment. This is a multicenter collaborative PD open‐source database that includes data such as neuropsychological scale, MRI, and genetic data. The NACC was established in 1999, it is a large‐scale compilation of longitudinal data for neurodegenerative diseases, including standardized clinical and neuropathological research data collected from Alzheimer's disease centers across the United States [12]. For information on the ethical review of the data, please refer to the website. A total of 272 patients from PPMI database who were diagnosed with PD at baseline were included in the study, 81 of whom developed DPD during the 5‐year follow‐up period. The 45 patients collected from the NACC database were used as an external validation set of unsupervised clustering method, of which 13 patients progressed to DPD during a 5‐year follow‐up period. The specific screening process can be found in Figure S1. The inclusion criteria were as follows: (1) all patients initially diagnosed with PD were followed up and (2) MRI examinations were performed, and complete clinical data were available. The exclusion criteria were as follows: (1) follow‐up < 5 years; (2) errors in the original MRI DICOM file that failed to extract the radiomic features; and (3) lack of specific clinical information. All patients underwent brain MRI, cerebrospinal fluid testing, and clinical symptom assessment at the initial assessment.

### 2.2 Radiomic Feature Preprocessing

All experimental data were obtained by 1.5T/3.0T MRI system scanning, including T1WI images of all patients. To further reduce the impact of image scanning parameters on feature extraction, we preprocessed structural image T1 images, including converting pixel values into 1 × 1 × 1 images. After the gray level was adjusted to 32, the preprocessed images were imported into the statistical parameter mapping SPM12 software of the MATLAB platform (version V2.5.5), and the images were automatically segmented into gray matter, white matter, and cerebrospinal fluid. The ITK‐SNAP package (http://www.itksnap.org/pmwiki/pmwiki.php) was used to manually correct segmented regions, including (1) removing nonbrain tissue, brainstem, and cerebellum and (2) correcting segmentation errors in brain tissue. The manual correction of the MR images was independently performed by two experienced neuroradiologists who were unaware of the clinical data. After manual correction, the segmented brain tissue regions were imported as masks into PyRadiomics software for feature extraction [13].

### 2.3 Radiomic Feature Extraction

The PyRadiomics platform was used to extract radiomic features related to digital image markers of the volume of interest (VOI). The extracted features include the following two categories: (1) original image features: ① 14 types of shape‐based features; ② 18 types of first‐order statistics; and ③texture features, including 22 types of gray‐level co‐occurrence matrix (GLCM) features, 16 types of gray‐level run‐length matrix (GLRLM) features, 16 types of gray‐level size zone matrix (GLSZM) features, and 14 types of gray‐level dependency matrix (GLDM) features; and (2) features related to the filter class: ① wavelet (WT) features and ② Laplacian transforms. A total of 3396 radiomic features were extracted, including 1132 gray matter (GM) features, 1132 white matter (WM) features, and 1132 cerebrospinal fluid (CFS) features. Detailed feature information can be found in Table S1. These features were extracted on the basis of the region of interest and manually modified by radiologists to obtain the most consistent features among different radiologists, thereby ensuring robustness. The correlation coefficients (CCs) of each feature between feature set A (from radiologist A) and feature set B (from radiologist B) were calculated using Spearman rank correlation. Features with CC > 0.8 were considered robust.

### 2.4 Unsupervised Cluster Analysis

First, the *z* score was used to normalize the extracted radiomic features, and the Supporting Information can be seen in detail. Principal component analysis (PCA) was used to perform feature reduction on the normalized radiomic features to reduce the dimension of the features and reduce the noise in the data. The PCA components that cumulatively accounted for 90% of the variance of the data were selected for the clustering step. In the clustering stage, we used four different clustering methods: Gaussian mixture model (GMM), hierarchical clustering, K‐means clustering (KMeans), and partitioning around medoids (PAM), the details of these four clustering methods can be found in the Supporting Information. For each clustering method, we determine the optimal number of clusters based on the silhouette width value and classify all cases into different subtypes accordingly. After that, Kaplan–Meier survival curve analysis was used to detect the time difference in PD progression to DPD among subtypes and compare the difference in the number of DPD cases among subtypes. Subtypes with rapid PD progression and a large number of DPDs were defined as the high‐risk subtype group. To further validate the generalization performance of the unsupervised clustering method, we applied the optimal clustering approach to the NACC dataset to evaluate whether it could also identify the high‐risk subtype group. Additionally, to substantiate the superiority of the high‐risk subtype model constructed using unsupervised cluster analysis, we developed a traditional model employing supervised logistic regression based on the entire PPMI dataset. Using the cut‐off value derived from the receiver operating characteristic (ROC) curve, we divided the dataset into two subgroups and compared whether there are differences in the progression times and the number of progression cases between the two subgroups.

### 2.5 Clinical Analysis of Cluster Subtypes

To determine the potential underlying substrates among the subtypes, the demographic characteristics (age, Gender, family genetic history, education), clinical symptoms (Hoehn–Yahr stages, Tremor‐dominant Parkinson's disease, Postural Instability and Gait Difficulty type Parkinson's disease, Other types of Parkinson's disease, Epworth Sleepiness Scale, Rapid Eye Movement Behavior Disorder, Mild Cognitive Impairment, Mental, Behavioral and Mood Disturbances Unified Parkinson's Disease Rating Scale, Activities of Daily Living Unified Parkinson's Disease Rating Scale, Motor Examination Unified Parkinson's Disease Rating Scale), cerebrospinal fluid levels (amyloid‐beta, alpha synuclein, microtubule‐associated protein tau, phosphorylated tau) and neuroimaging characteristics (standardized white matter, gray matter, and cerebrospinal fluid volumes) among the subtypes were compared and analyzed.

### 2.6 Construction of Subtype Prediction Model

Considering that the specific clinical characteristics and disease progression modalities of high‐risk subtypes might possess distinctive biological foundations, this study was directed toward establishing predictive models for high‐risk subtypes. High‐risk subtype group was randomly divided into training subgroups and testing subgroups at a 7:3 ratio. Multivariate logistic regression was used to determine the independent risk factors for the training subgroup data, and a prediction model using a decision tree was built on the basis of the above independent risk factors to identify high‐risk PD patients. The cross‐validation process of decision tree (DT) algorithm based on training queue includes two nested loops: one outer loop has a repeated hierarchical random segmentation training queue consisting of a training subset and a testing subset, in which 100 repetitions are used to evaluate the classification performance, and the other inner loop has fivefold cross‐validation, which is used to optimize the hyper parameters of the algorithm, and the selection of hyperparameters was automated using the GridSearchCV toolkit. One model is created for each segmentation, resulting in 100 models. We utilized the receiver operating characteristic (ROC) curve and its area under the curve (AUC) to evaluate the performance of the predictive model within the testing subset. Additionally, the average AUC value of 100 iterations was calculated as a reliability evaluation of the model performance. The details of cross‐validation can be found in the Supporting Information.

### 2.7 Validation of Predictive Model

**FIGURE 1:**
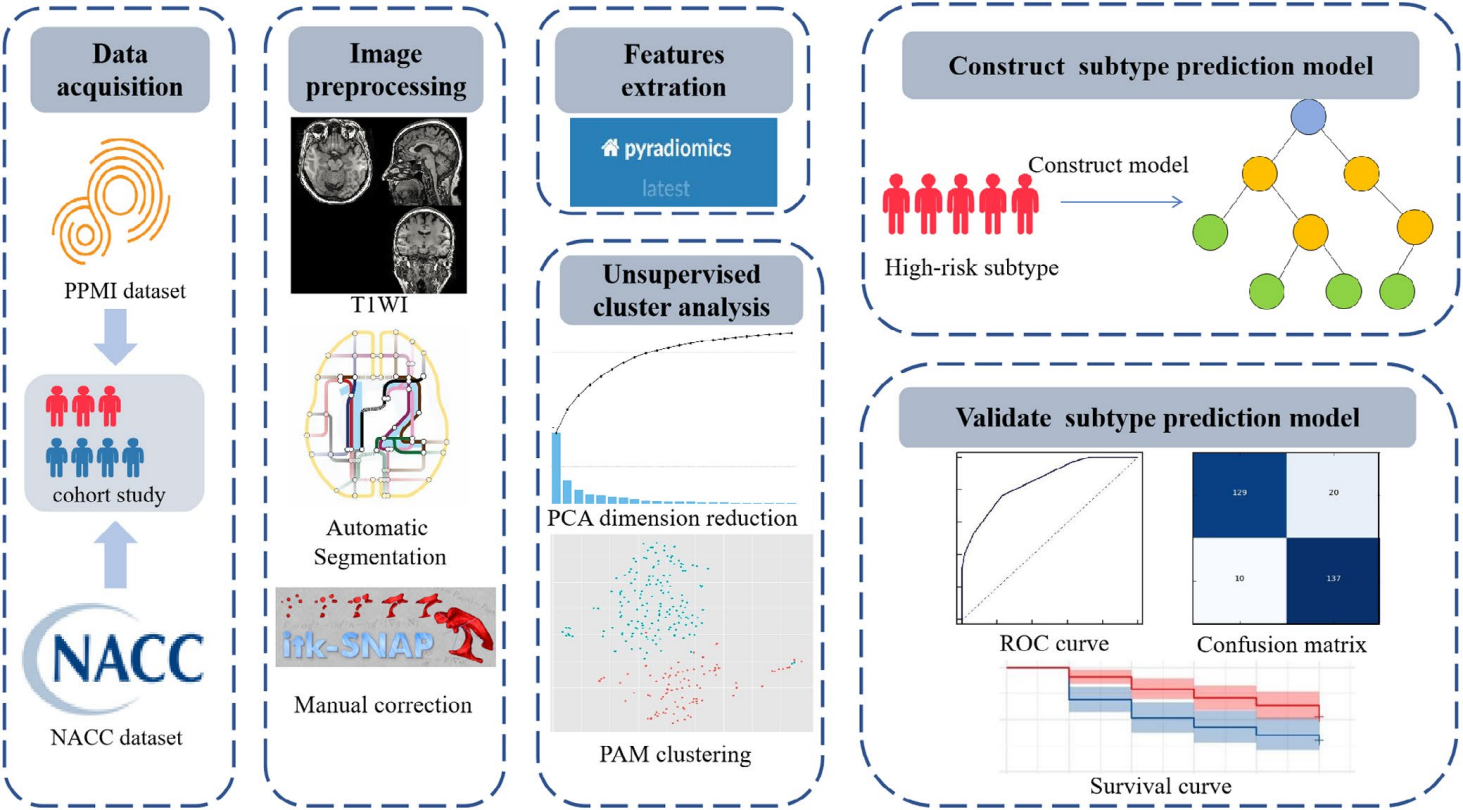
Architecture of unsupervised machine learning using whole brain radiomics analysis to identify depression subtypes in the progression of PD.

To verify the accuracy of the model, we evaluated its performance in both training and testing subgroups. Additionally, we applied the model to various subtype groups to assess its generalization performance. We also examined the goodness of fit for the joint model using the Hosmer–Lemeshow test and utilized calibration curves to visually examine the concordance between predicted DPD probabilities and actual DPD probabilities. Furthermore, to evaluate differences between high‐risk subtype predictions and overall cohort predictions, we constructed a traditional supervised logistic regression model based on high‐risk subtypes identified through unsupervised clustering. The performance of this model was then compared with that of a traditional model developed using data from the entire PPMI cohort. The specific process is illustrated in Figure 1.

### 2.8 Statistical Analysis

All the statistical analyses were conducted using R statistical software (v. 3.5.1), MedCalc software (V.11.2; 2011 MedCalc software bvba, Mariakerke, Belgium), and SPSS software 25.0 (IBM, Armonk, NY). The Kolmogorov–Smirnov test was used to evaluate the normality of the variable distribution. If the distribution was normal, an independent‐sample t test was used to assess group differences and continuous variables for normal distribution were presented as the mean ± standard deviation. For data without a normal distribution, the Mann–Whitney *U* test was used, and variables were expressed as medians (interquartile ranges, IQRs). For classified data, Categorical variables were recorded as frequencies (%), and the chi‐squared test or Fisher's exact test was used to evaluate the association of categorical variables. All of the statistics are bidirectional, and a *p* value of less than 0.05 is considered statistically significant.

## 3 Results

### 3.1 Unsupervised Machine Learning for Radiomics

**FIGURE 2:**
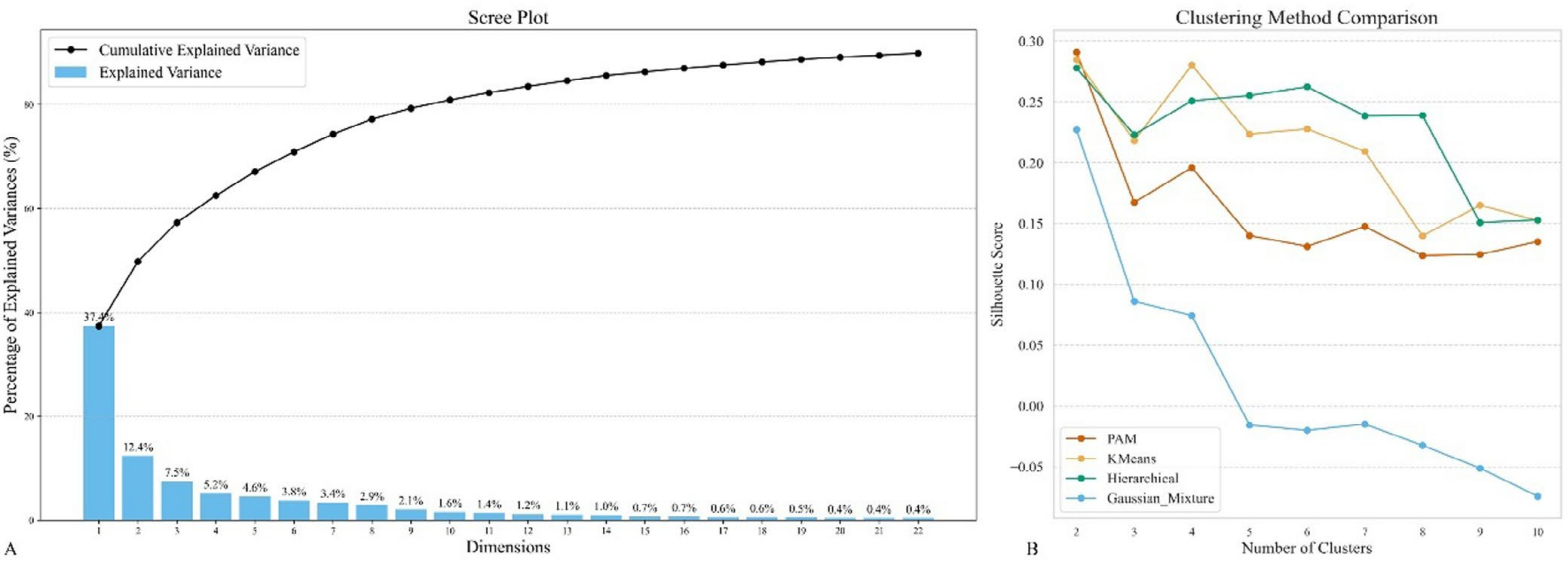
(A) Illustrates the display of PCA feature weights, highlighting 22 features that account for 90% of the variability within the feature space. (B) Presents a comparative analysis of four clustering methods based on classification quantity, revealing that PAM demonstrates the highest silhouette coefficient and achieves superior performance.

**FIGURE 3:**
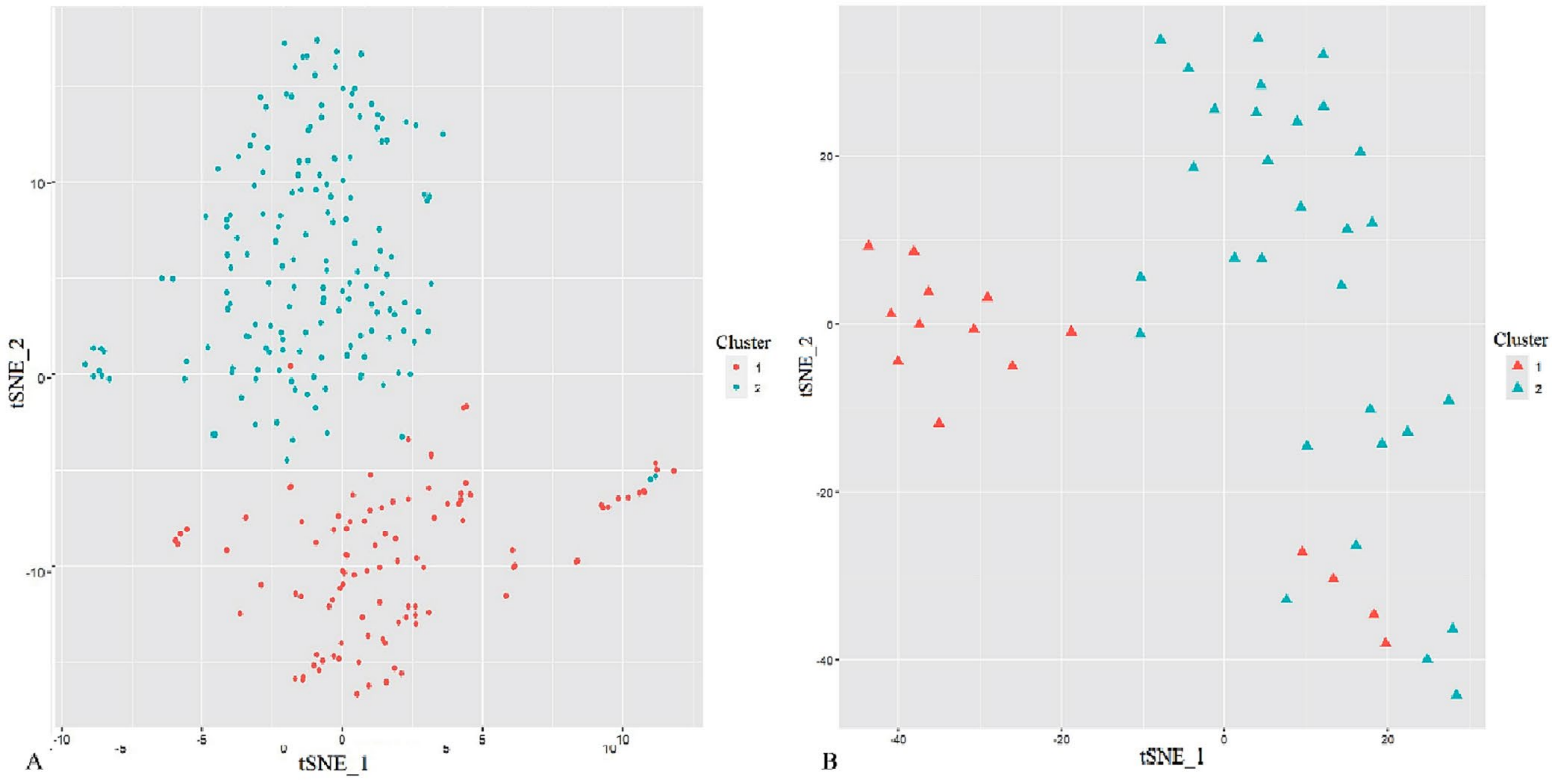
(A and B) Illustrate the visualization of clustering results derived from PCA principal components in the PPMI dataset and NACC dataset, respectively. In these figures, each point represents an individual case, while distinct colors denote different subgroups. The horizontal axis corresponds to the first principal component, whereas the vertical axis represents the second principal component.

PCA generated 22 principal components, explaining 90% of the variability in the feature space. Through an unsupervised clustering analysis of these 22 principal components, it was determined that PAM exhibits the highest silhouette coefficient and achieves optimal performance when the number of clusters is set to two. Specifically, with two clusters, PAM's silhouette coefficient surpasses those of the other three methods evaluated. Notably, PAM not only demonstrates the highest silhouette coefficient at this cluster count but also maintains robust stability as the number of clusters increases. Based on these findings, PAM was selected as the most effective clustering method for this study, as illustrated in Figure 2. Then, based on the closest distance to the two identified cluster centers, cluster labels were assigned to each subject in the PPMI dataset. Clusters 1 and 2 both comprised 136 subjects, and these two clusters were utilized for comparing DPD status. Additionally, cluster labels were also assigned to each subject in the NACC dataset, with Cluster 1 containing 14 subjects and Cluster 2 containing 31 subjects. The scatter plots of the clusters from both datasets visually represent the clustering results, as illustrated in Figure 3.

### 3.2 Comparison of Relevant Characteristics Based on Subtype

There were statistically significant differences in mild cognitive impairment (MCI) status, years of education, and quantitative values of abeta between the two clustered subgroups from PPMI dataset (*p* < 0.05), in which the number of MCI cases in cluster subgroup 1 was significantly greater than that in cluster subgroup 2, whereas the quantitative values of years of education and abeta were significantly lower than those in cluster subgroup 2. The other demographic and clinical characteristics were not significantly different (*p* > 0.05), as shown in Table 1.

**TABLE 1:** Comparative analysis of relevant features among cluster subgroups.

Variable		Sample	Cluster subtypes 1	Cluster subtypes 2	*p*
Age		272	61.50 ± 10.17	61.85 ± 9.37	0.767
Gender	Male	177	91 (66.91%)	86 (63.24%)	0.525
	Female	95	45 (33.09%)	50 (36.76%)	
EDUCYRS		272	16.0 (13.0, 18.0)	16.0 (14.0, 18.0)	0.02*
Family genetic history	No	71	40 (29.41%)	31 (22.79%)	0.214
	Yes	201	96 (70.59%)	105 (77.21%)	
H‐Y stages	0	9	5 (3.68%)	4 (2.94%)	0.565
	1	120	63 (46.32%)	57 (41.91%)	
	2	141	68 (50.00%)	73 (53.68%)	
	3	2	0 (0.00%)	2 (1.47%)	
PD subtype	TD	193	93 (68.38%)	100 (73.53%)	0.635
	PIGD	43	23 (16.91%)	20 (14.71%)	
	Other	36	20 (14.71%)	16 (11.76%)	
ESS	No	239	121 (88.97%)	118 (86.76%)	0.577
	Yes	33	15 (11.03%)	18 (13.24%)	
RBD	No	172	86 (63.24%)	86 (63.24%)	1.0
	Yes	100	50 (36.76%)	50 (36.76%)	
MCI	No	231	108 (79.41%)	123 (90.44%)	0.011*
	Yes	41	28 (20.59%)	13 (9.56%)	
UPDRS3_score		272	18.0 (12.0, 24.0)	20.0 (15.0, 25.0)	0.106
UPDRS1_score		272	5.0 (2.0, 7.0)	5.0 (3.0, 7.0)	0.605
UPDRS2_score		272	4.0 (2.0, 8.0)	4.0 (2.0, 7.0)	0.978
abeta		272	872.25 (663.15, 1132.25)	944.80 (705.35, 1290.75)	0.048*
asyn		272	1465.20 (1154.18, 1947.28)	1443.30 (1132.83, 1934.88)	0.927
tau		272	160.80 (137.03, 209.63)	164.15 (137.40, 214.73)	0.994
ptau		272	13.44 (11.29, 17.69)	13.77 (11.22, 18.04)	0.973
GM volume		272	2.39 (2.37, 2.43)	2.38 (2.37, 2.43)	0.726
WM volume		272	2.81 ± 0.20	2.77 ± 0.22	0.175
CSF volume		272	4.43 (4.50, 4.82)	4.63 (4.57, 4.87)	0.412

Abbreviations: CSF, cerebrospinal fluid; EDUCYRS, categorical education; Ess, Epworth Sleepiness Scale; GM, gray matter; H‐Y stages, Hoehn–Yahr stages; MCI, mild cognitive impairment; PIGD, postural instability/gait disorder; RBD, rapid‐eye‐movement behavior disorder; TD, tremor dominate; UPDRS, Unified Parkinson's Disease Rating Scale; WM, white matter.

* *p* < 0.05.

### 3.3 Determination and Validation of High‐Risk Subtypes

**FIGURE 4:**
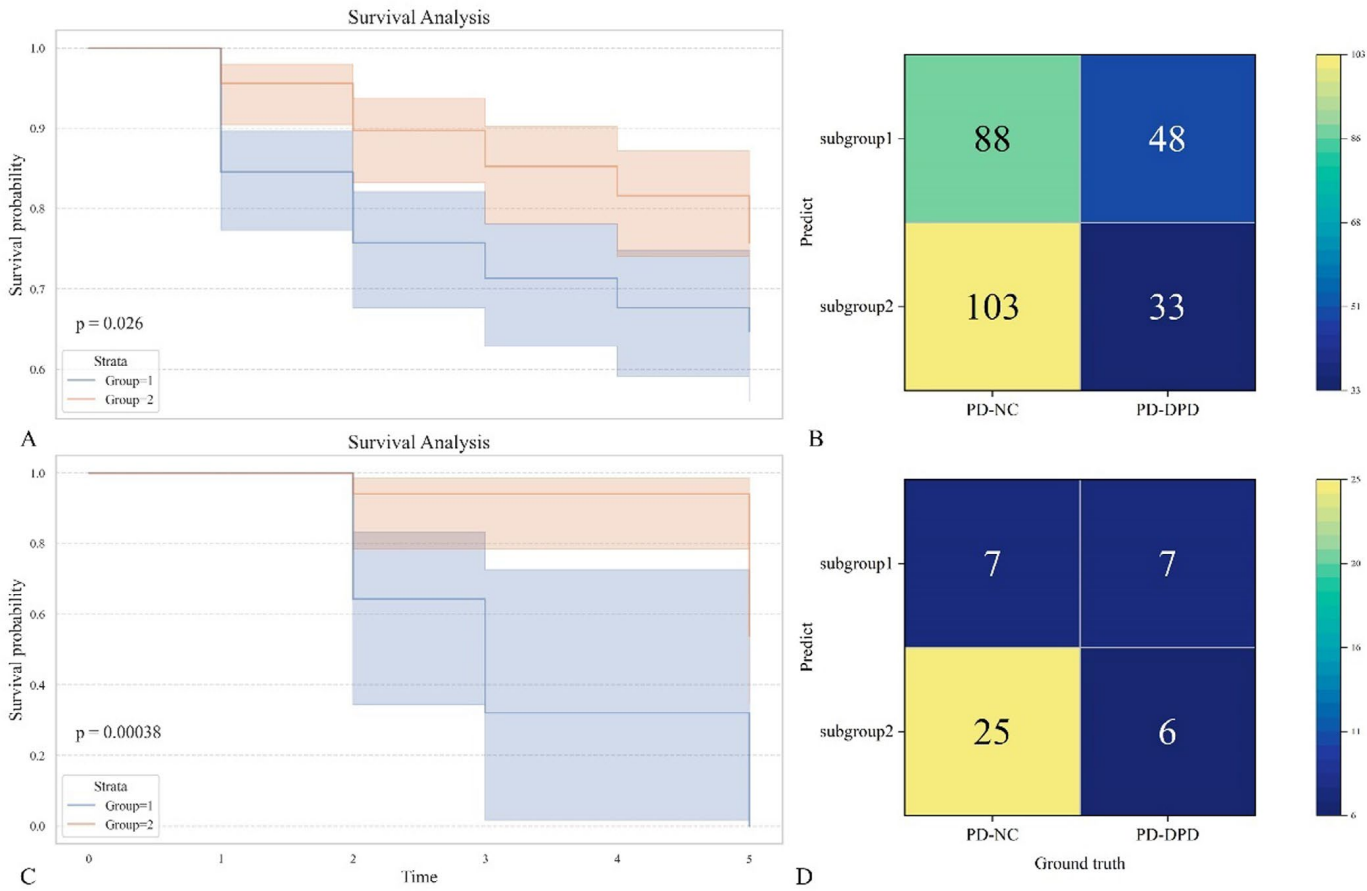
(A and C) Demonstrate a significant difference in the progression time of PD between the two cluster subgroups derived from the PPMI and NACC datasets. The confusion matrix plot presented in B and D illustrates the differences in the distribution of DPD cases between the two subgroups derived from the PPMI and NACC datasets.

Kaplan–Meier survival curve analysis revealed that there was a significant difference in DPD progression time between the two cluster subgroups from PPMI dataset (*p* < 0.05); the mean progression time in subgroup 1 was 3.99 years, and that in subgroup 2 was 4.52 years. On the other hand, there was also a significant difference in the actual number of DPD cases among the subgroups on the basis of clustering (*p* < 0.05), where the number of DPD cases in subgroup 1 was 48 and that in subgroup 2 was 33. On the basis of the above results, subtype 1 was defined as a high‐risk subtype group in this study. A logistic regression analysis conducted on the high‐risk subtype group indicated that RBD, UPDRS I score, UPDRS II score, and ptau serve as independent predictors of DPD. For further details, please refer to Table 2. Finally, unsupervised clustering methods were validated on the NACC dataset, revealing a significant difference in DPD progression time between the two cluster subgroups (*p* < 0.05). The mean progression time for subgroup 1 was found to be 2.28 years, while that for subgroup 2 was 4.69 years. Additionally, there was a notable difference in the actual number of DPD cases between these subgroups (*p* < 0.05), with subgroup 1 comprising seven cases and subgroup 2 consisting of six cases, as illustrated in Figure 4. In addition, based on data from the PPMI full cohort, logistic regression analysis identified PD subtype, UPDRS1 score, ptau levels, and GM volume as independent predictors; detailed results are presented in Table S3. A supervised logistic regression model constructed using these independent predictors classified patients into two subgroups based on a cut‐off value of 0.42019. The first subgroup included 50 individuals, among whom 15 had DPD, while the second subgroup comprised 222 individuals with DPD present in 66 cases. No significant difference was observed in the number of DPD cases between these two subgroups (*p* > 0.05). The progression times for the two subgroups were 4.23 and 4.34 years, respectively, and there was also no significant difference (*p* > 0.05).

**TABLE 2:** Multifactor logistic regression analysis based on clustering high‐risk subgroups.

Variable	Cluster subtypes 1			
	Univariate analysis		Multivariate analysis	
	OR	*p*	OR	*p*
Age	1.020 (0.984, 1.057)	0.287	NA	NA
Gender	1.562 (0.747, 3.267)	0.236	NA	NA
EDUCYRS	0.934 (0.833, 1.048)	0.248	NA	NA
Family genetic history	1.400 (0.634, 3.093)	0.405	NA	NA
Hoehn–Yahr stages	1.384 (0.827, 3.952)	0.987	NA	NA
PD subtype	2.128 (0.839, 5.396)	0.112	NA	NA
UPDRS1	1.211 (1.077, 1.361)	0.001*	1.160 (1.018, 1.322)	0.026*
UPDRS2	1.157 (1.055, 1.270)	0.002*	1.111 (1.000, 1.233)	0.049*
UPDRS3	1.014 (0.977, 1.053)	0.459	NA	NA
ESS	1.707 (0.579, 5.038)	0.333	NA	NA
RBD	2.079 (1.006, 4.293)	0.048*	2.383 (1.058, 5.370)	0.036*
abeta	1.000 (0.999, 1.001)	0.520	NA	NA
asyn	1.000 (1.000, 1.001)	0.244	NA	NA
tau	1.009 (1.002, 1.015)	0.007*	NA	NA
ptau	1.097 (1.026, 1.173)	0.006*	1.102 (1.021, 1.189)	0.013*
MCI	1.808 (0.776, 4.208)	0.170	NA	NA
GM volume	2.166 (0.370, 12.687)	0.392	NA	NA
WM volume	3.144 (0.509, 19.406)	0.217	NA	NA
CSF volume	0.864 (0.587, 1.271)	0.457	NA	NA

*Note:* NA, not available because the variable is not included in multiple variables. The *p* value indicates whether the variable is an independent predictor of PD‐MCI.

Abbreviations: CSF, cerebrospinal fluid; EDUCYRS, Categorical Education; Ess, Epworth Sleepiness Scale; GM, gray matter; MCI, mild cognitive impairment; PIGD, postural instability/gait disorder; RBD, rapid‐eye‐movement behavior disorder; TD, tremor dominate; UPDRS, Unified Parkinson's Disease Rating Scale; WM, white matter.

* *p* < 0.05.

### 3.4 Construction and Validating of a DPD Prediction Model on the Basis of Cluster Subtypes

**FIGURE 5:**
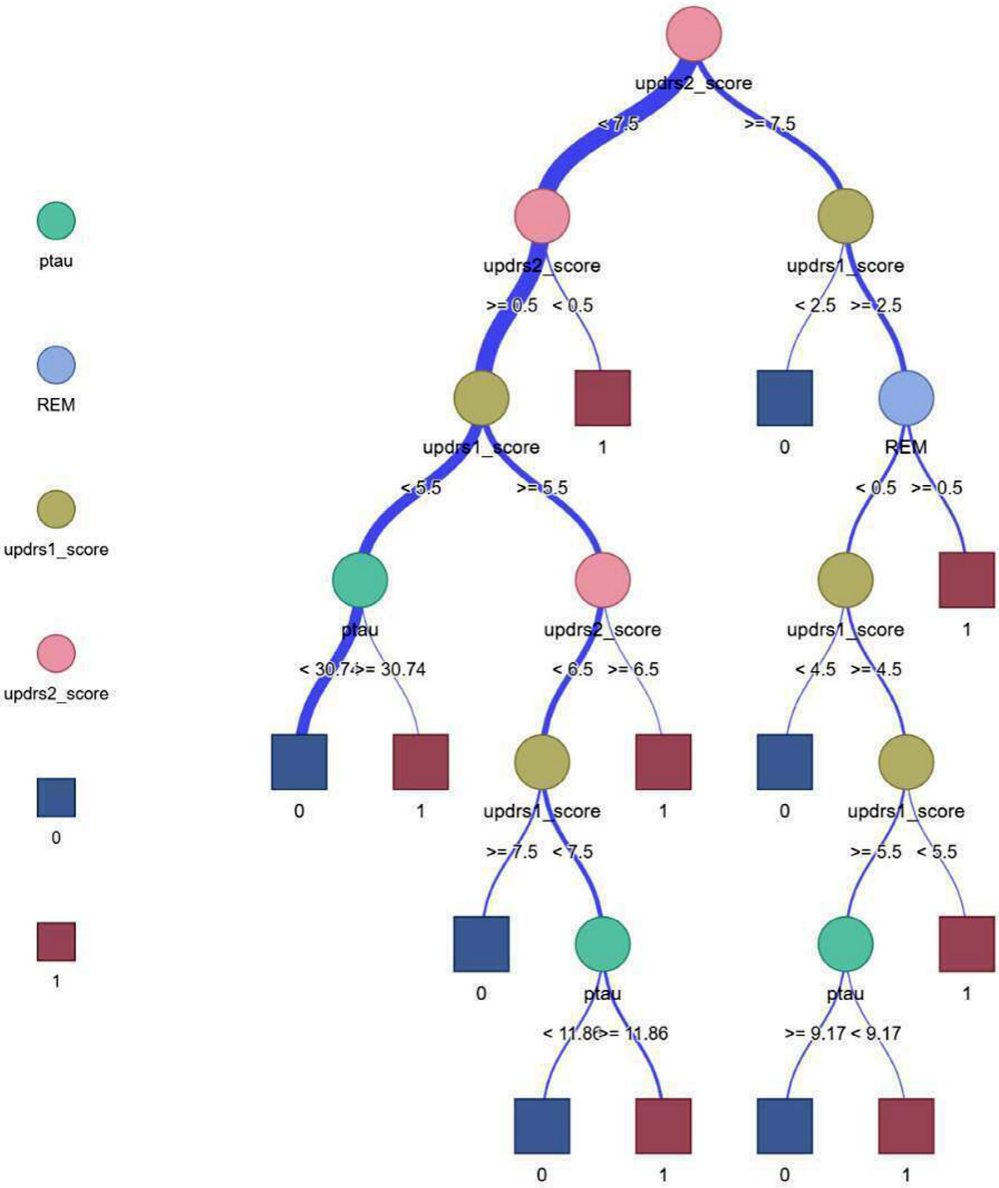
Visualization of the Tree model, where the square frame represents the clinical outcome. In this study, a probability value of 1 represents that PD patients progress to DPD, and a probability value of 0 represents that PD patients do not progress to DPD.

**FIGURE 6:**
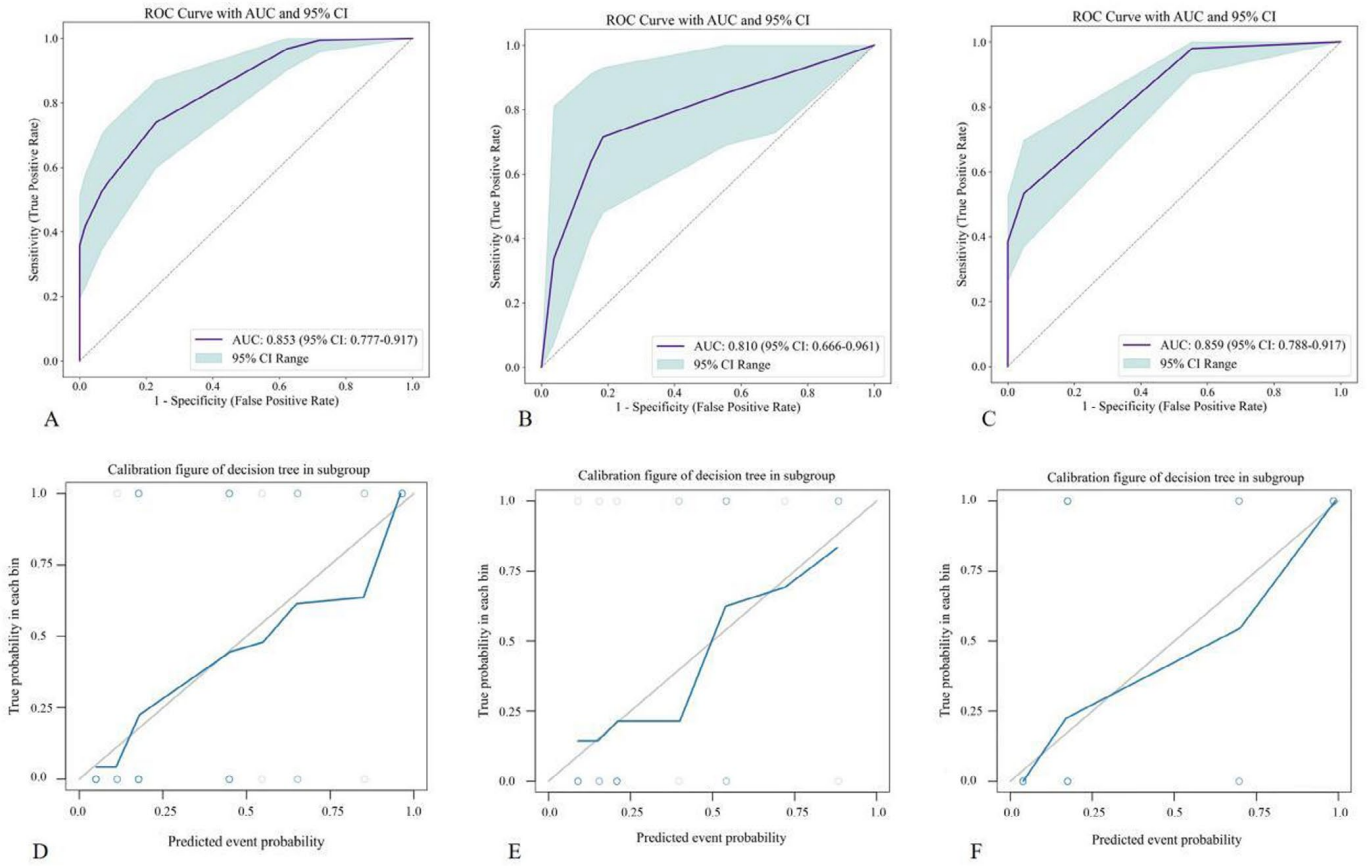
(A–C) Show the diagnostic performance of the model in the training subgroup, testing subgroup, and clustering subgroup 2, respectively; (D–F) Show the calibration figure of decision tree in the training subgroup, testing subgroup, and clustering subgroup 2, respectively.

A traditional model was constructed using logistic regression based on independent predictive factors of high‐risk subgroups. The AUC of the traditional model was 0.731. Subsequently, DT algorithm was used to construct a machine learning model, the average AUC value of 100 iterations was calculated as 0.825, and indicates the stability of the DT model, as illustrated in Figure 5. The Hosmer–Lemeshow test revealed that the DT model did not overfit (*p* > 0.05), and the correction curve revealed that the prediction efficiency of the DT model was consistent with that of the actual DPD state. The AUC values of the model in the training and testing subgroups were 0.853 and 0.81, the sensitivities were 0.765 and 0.786, and the specificities were 0.771 and 0.815, respectively. To test the generalization performance of the DT model, we also applied the model to clustering subgroup 2, whose AUC, sensitivity and specificity were 0.859, 0.654, and 0.852, respectively, as shown in Figure 6. In contrast, the traditional logistic regression model based on data from the PPMI cohort exhibited a lower AUC value of only 0.674 compared to that obtained from our traditional high‐risk subgroup model.

## 4 Discussion

The results of this study show that cluster analysis of whole‐brain radiomic features can identify high‐risk subtypes of DPD without any prior classification. The main advantage of this method is that it can reveal relatively homogeneous subtypes from a large number of samples in an impartial manner, which may also indicate that MRI whole‐brain radiomics contain DPD‐related information. Furthermore, when compared to traditional supervised logistic regression classification methods, our results demonstrate that unsupervised clustering analysis can more accurately identify high‐risk subtypes, which highlights the sensitivity of unsupervised clustering. On the other hand, our results also show that the predictive model built on the basis of cluster subgroups has good diagnostic efficacy in identifying DPD patients in both high‐risk and low‐risk subgroups, while the model built on the full cohort has lower diagnostic performance compared to the subgroup‐based predictive models. This observation implies that when developing models across the overall participant population, the distinctive characteristics associated with high‐risk subtypes may be obscured by features from other subtypes. In contrast, modeling specifically for high‐risk groups allows for the capture of subtype‐specific risk factors, thereby enhancing prediction accuracy for these particular subtypes. Such insights could prove invaluable in advancing our understanding of DPD and improving our capacity to formulate comprehensive diagnoses.

A major and common criticism of unsupervised tasks is the difficulty in interpreting the clinical significance of their classification [14]. However, in this study, we addressed these questions by analyzing subtypes' clinical symptoms, clinical scale scores, and demographic information. MCI status, years of education, and abeta quantitative values were significantly different across the two cluster subtypes we studied. Specifically, the number of MCI cases in the high‐risk subgroup was significantly greater than that in the nonhigh‐risk subgroup, whereas years of education and abeta quantitative values were significantly lower in the high‐risk subgroup. These differences suggest that these factors may work together to increase an individual's risk of developing DPD. In fact, previous studies have shown that the educational level of depressed patients is generally lower [15], which is also consistent with the results of this study. The difference in years of education may reflect the difference in cognitive reserve, and individuals with fewer years of education may be more susceptible to a decline in cognitive function [16]. On the other hand, differences in abeta quantitative values may also be related to the pathological mechanism of Alzheimer's disease, and a lower abeta level may reflect a more severe pathological state [17]. Interestingly, both AD and major depressive disorder (MDD), the disease endpoints of MCI progression, are highly prevalent neuropsychiatric disorders with epidemiological overlap [18]. Therefore, the above research results also suggest that some factors related to cognitive status in PD patients may affect the process of their conversion to DPD, which is similar to the findings of Ng et al. [19], who reported that there is a correlation between DPD and cognitive decline.

We also analyzed independent risk factors in high‐risk cluster subgroups and found that REM, the UPDRS1 score, the UPDRS2 score, and ptau protein in the high‐risk subgroup were independent predictors of DPD, suggesting the importance of these indicators in clinical prediction. In particular, sleep behavior disorders are considered to be the most representative factor in predicting the risk of depression [20] and are highly correlated with depression [21]. REM sleep behavior disorder (RBD) is very common in patients with Parkinson's disease. RBD is not only an early marker of Parkinson's disease but also closely related to patients' depressive symptoms. Gu et al. [22] showed that REM is an important predictor for patients with DPD, consistent with the results of this study. RBD is related to damage to neurons in the brainstem, which also regulates emotional and cognitive functions, and this damage may lead to depressive symptoms [23], which may also be one of the possible reasons for the involvement of RBD in the progression of DPD. Therefore, we speculate that these lesions may affect emotional regulation and induce Interestingly, in our study, tau was an important predictor of DPD, rather than abeta, as previously mentioned, although it differed significantly between the high‐risk and nonhigh‐risk subgroups. Tau protein is a type of microtubule‐associated protein, and the accumulation of p‐tau protein leads to the formation of neurofibrillary tangles, impairs the function of neurons, and leads to neurodegeneration depression [24]. In fact, tau protein misfolding and aggregation are pathological markers of Alzheimer's disease and more than 20 other neurodegenerative diseases [25], which is also consistent with the conclusion of our analysis above that cognitive status is closely related to DPD.

On the other hand, our study also revealed that UPDRS1 and − 2 scores are independent predictors of DPD. A meta‐analysis revealed that the UPDRS can identify DPD with a sensitivity of up to 0.72 and a specificity of up to 0.80 [26], and other studies have shown that UPDRS1 can be used as a screening tool for depression [27]. Our research results also confirmed the above conclusion: UPDRS1 reflects the severity of a patient's emotional disorder, and a higher UPDRS1 score indicates that the patient's emotional state is poor and a poor emotional state is more likely to lead to depression [28], which further confirmed that UPDRS1 may reflect some internal states of DPD. The UPDRS2 score reflects the independence and activity ability of patients in daily life, which in turn indirectly reflects the pathological status of PD. These pathological changes in specific brain areas may also damage emotional function and lead to depression [29].

In this study, we also built a DPD prediction model based on the clustering subtype via a decision tree algorithm, and the model achieved high prediction efficiency and stability and high diagnostic performance for low‐risk subtypes. These findings indicate that the model has strong generalizability and can be applied to different subtypes of DPD patients. In fact, similar studies have been conducted to predict DPD. Li et al. [30] used plasma biomarkers combined with MRI radiomics features to construct a model to predict DPD, and the diagnostic accuracy reached 0.937, which is significantly higher than our results; however, the acquisition of plasma biomarkers is an invasive examination that may not be suitable for universal screening. In another study, Byeon integrated a variety of risk factors, including demographic characteristics, psychiatric scale scores, clinical characteristics, disease grade, etc., and built a prediction model using SVM; its diagnostic performance reached 0.925 [31]. Although it also has advantages over the results of this study, the decision tree model used in this study has visual diagrams. This method is convenient for clinical application. Overall, the unsupervised cluster analysis of high‐risk groups used in this study may not perform as well as the supervised classification model, but it may be easier to obtain relevant screening results at a low cost in clinical practice.

There are several limitations to this study. First, this study was designed as a retrospective analysis, which may have resulted in selection bias and information bias. Future research should adopt a prospective longitudinal study design to gain a more comprehensive understanding of the progression from PD to DPD. Second, the research sample mainly comes from specific public datasets, which may not represent a broader population of PD patients. Future studies should consider expanding the sample size and increasing the diversity of the sample, including participants from different regions, races, and cultural backgrounds, to improve the universality and generalizability of the results. Finally, the follow‐up time used in this study may not have been sufficient to fully capture the long‐term progression of PD patients to DPD. A longer follow‐up time may reveal more details about disease progression, but as a conceptual study, it provides a strong theoretical basis for the transformation of DPD.

In conclusion, this study subdivided the subtypes of DPD patients by unsupervised cluster analysis, revealed the differences in the clinical characteristics and time to progression across different subtypes, and constructed an effective DPD prediction model. These results not only provide a theoretical basis for the individualized diagnosis and treatment of DPD patients but also provide a powerful tool for clinical practice.

## Author Contributions

Z.Z. and J.P. conceptualized the study. Q.S. collected the clinical data. Y.X. collected the MRI data. J.P. and Y.W. analyzed the data. Z.Z. wrote the manuscript. Z.S. designed and supervised the whole research. All authors have read and agreed to the published version of the manuscript.

## Ethics Statement

The case data used in this study came from the Parkinson's Progression Markers Initiative (PPMI) (http://www.PPMI‐info.org) database, and data collection was approved by the institutional review board; For ethical review information on the data, please refer to the website.

## Conflicts of Interest

The authors declare no conflicts of interest.

## Supporting information


Data S1.

## Data Availability

The data that support the findings of this study are available from the corresponding author upon reasonable request.
