# Supplementary material for: Identification of Depression Subtypes in Parkinson's Disease Patients via Structural MRI Whole‐Brain Radiomics: An Unsupervised Machine Learning Study
Source: CNS Neurosci Ther. 2025 Feb 6;31(2):e70182. doi: 10.1111/cns.70182 (PMC11802460; doi:10.1111/cns.70182)
Supplement: Supplementary file 1 — Data S1. [file CNS-31-e70182-s001.docx]

**Supplementary material**

**1.Case inclusion and demographic information**

The case data included in this study came from the Parkinson's Progression Markers Initiative (PPMI) (http://www.PPMI-info.org) and National Alzheimer's Coordinating Center (NACC) (https://naccdata.org) dataset. According to the inclusion and exclusion criteria, 272 cases from the PPMI database and 45 cases from the NACC database were collected, the detailed process is shown in Figure S1. In addition, we also present information from both databases, as shown in Table S1.

**
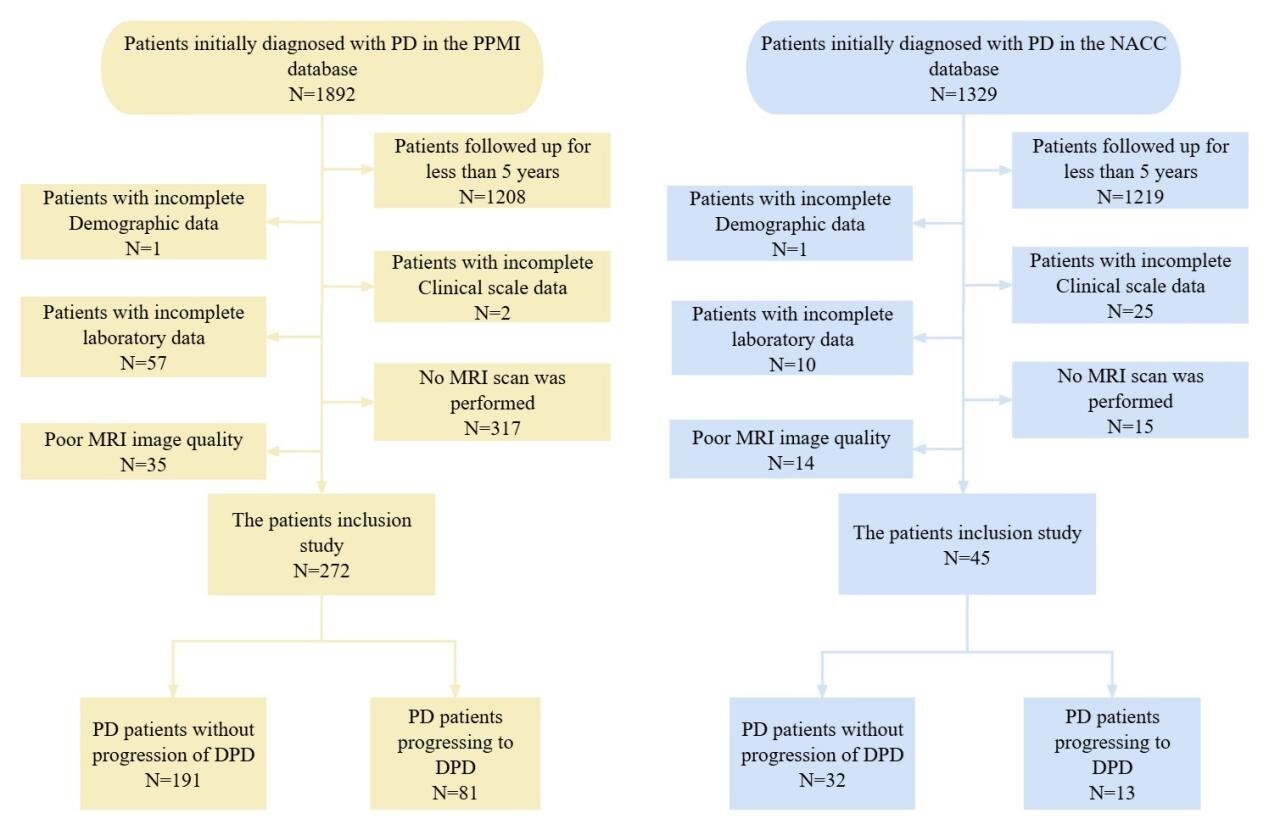
**

**Figure S1.** Inclusion and Exclusion Flowchart for the PPMI and NACC Databases

**Table S1. Demographic information of the PPMI Database and NACC Database**

| Variable | | PPMI  (n=272) | NACC  (n=45) |
| --- | --- | --- | --- |
| Age | | 61.68±9.76 | 73.89±6.73 |
| EDUCYRS | | 16 (15.36, 16.06) | 16 (14.81, 16.43) |
| Sex | Male | 177(82.71%) | 37 (17.29%) |
|  | Female | 95 (92.23%) | 8 (7.77%) |
| Fam | NO | 71 (78.02%) | 20 (21.98%) |
|  | YES | 201 (70.59%) | 25 (77.21%) |
| ESS | NO | 239 (89.85%) | 27 (10.15%) |
|  | YES | 33 (64.71%) | 18(35.29%) |
| MCI | NO | 231 (91.30%) | 22 (8.70%) |
|  | YES | 41 (64.06%) | 23 (35.94%) |

**Note:** EDUCYRS: Categorical Education; Fam: Family genetic history; MCI: Mild cognitive impairment.

**2.Standardization of data**

This study included a total of 1132 feature types, and detailed feature information can be found in Table S2. The extracted features were standardized before dimension reduction, which removed the unit limits of the data of each feature and converted it into a dimensionless pure value. This allowed the indices of different units or orders to be compared and weighted. We used Z-score normalization to make the feature intensities fit a standard normal distribution with μ and σ, wherein μ is the mean value of the features and σ is the standard deviation. The normalized values (also called Z-scores) of the feature intensities (x) were calculated as follows:


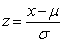


**Table S2. The information of radiomics features**

| Feature Groups (N) | Feature names | Feature Groups (N) | Feature names |
| --- | --- | --- | --- |
|  | firstorder_10Percentile |  | glszm_GrayLevelNonUniformity |
|  | firstorder_90Percentile |  | glszm_GrayLevelNonUniformityNormalized |
|  | firstorder_Energy |  | glszm_GrayLevelVariance |
|  | firstorder_Entropy |  | glszm_HighGrayLevelZoneEmphasis |
|  | firstorder_InterquartileRange |  | glszm_LargeAreaEmphasis |
|  | firstorder_Kurtosis |  | glszm_LargeAreaHighGrayLevelEmphasis |
|  | firstorder_Maximum |  | glszm_LargeAreaLowGrayLevelEmphasis |
| First-order features (N = 18) | firstorder_MeanAbsoluteDeviation | GLSZM texture features | glszm_LowGrayLevelZoneEmphasis |
|  | firstorder_Mean | (N = 16) | glszm_SizeZoneNonUniformity |
|  | firstorder_Median |  | glszm_SizeZoneNonUniformityNormalized |
|  | firstorder_Minimum |  | glszm_SmallAreaEmphasis |
|  | firstorder_Range |  | glszm_SmallAreaHighGrayLevelEmphasis |
|  | firstorder_RobustMeanAbsoluteDeviation |  | glszm_SmallAreaLowGrayLevelEmphasis |
|  | firstorder_RootMeanSquared |  | glszm_ZoneEntropy |
|  | firstorder_Skewness |  | glszm_ZonePercentage |
|  | firstorder_TotalEnergy |  | glszm_ZoneVariance |
|  | firstorder_Uniformity |  |  |
|  | glrlm_GrayLevelNonUniformity |  | glcm_Autocorrelation |
|  | glrlm_GrayLevelNonUniformityNormalized |  | glcm_ClusterProminence |
|  | glrlm_GrayLevelVariance |  | glcm_ClusterShade |
|  | glrlm_HighGrayLevelRunEmphasis |  | glcm_ClusterTendency |
|  | glrlm_LongRunEmphasis |  | glcm_Contrast |
| GLRLM texture features | glrlm_LongRunHighGrayLevelEmphasis | GLCM texture features | glcm_Correlation |
| (N = 16) | glrlm_LongRunLowGrayLevelEmphasis | (N = 24) | glcm_DifferenceAverage |
|  | glrlm_LowGrayLevelRunEmphasis |  | glcm_DifferenceEntropy |
|  | glrlm_RunEntropy |  | glcm_DifferenceVariance |
|  | glrlm_RunLengthNonUniformity |  | glcm_Id |
|  | glrlm_RunLengthNonUniformityNormalized |  | glcm_Idm |
|  | glrlm_RunPercentage_T2 |  | glcm_Idmn |
|  | glrlm_RunVariance |  | glcm_Idn |
|  | glrlm_ShortRunEmphasis |  | glcm_Imc1 |
|  | glrlm_ShortRunHighGrayLevelEmphasis |  | glcm_Imc2 |
|  | glrlm_ShortRunLowGrayLevelEmphasis |  | glcm_InverseVariance |
|  |  |  | glcm_JointAverage |
|  |  |  | glcm_JointEnergy |
|  |  |  | glcm_JointEntropy |
|  |  |  | glcm_MCC |
|  |  |  | glcm_MaximumProbability |
|  |  |  | glcm_SumAverage |
|  |  |  | glcm_SumEntropy |
|  |  |  | glcm_SumSquares |
|  | gldm_DependenceEntropy |  | ngtdm_Busyness |
|  | gldm_DependenceNonUniformity |  | ngtdm_Coarseness |
| GLDM texture features | gldm_DependenceNonUniformityNormalized | NGTDM texture features | ngtdm_Complexity |
| (N = 14) | gldm_DependenceVariance | (N = 5) | ngtdm_Contrast |
|  | gldm_GrayLevelNonUniformity |  | ngtdm_Strength |
|  | gldm_GrayLevelVariance |  |  |
|  | gldm_HighGrayLevelEmphasis |  |  |
|  | gldm_LargeDependenceEmphasis |  |  |
|  | gldm_LargeDependenceHighGrayLevelEmphasis |  |  |
|  | gldm_LargeDependenceLowGrayLevelEmphasis |  |  |
|  | gldm_LowGrayLevelEmphasis |  |  |
|  | gldm_SmallDependenceEmphasis |  |  |
|  | gldm_SmallDependenceHighGrayLevelEmphasis |  |  |
|  | gldm_SmallDependenceLowGrayLevelEmphasis |  |  |
| Los features (N = 186) | Log-sigma-1.0，2.0_* (N =186) |  |  |

Note: GLCM, Gray-level co-occurrence matrices; GLRLM, Gray-level run length matrix; GLSZM, Gray-level size zone matrix; GLDM, Gray-level dependence matrix. *The abbreviated representation of feature types

**3.Principal component analysis:**

Principal Component Analysis (PCA) is a commonly used data dimensionality reduction technique used to transform a set of potentially correlated variables into a set of linearly uncorrelated variables through orthogonal transformation, known as principal components.

**Principle**: The purpose of PCA is to identify the main components in the dataset that can explain the maximum variance in the data. The steps usually include:

**Calculate covariance matrix**: The covariance matrix describes the correlation between various features in the dataset.

**Solving eigenvalues and eigenvectors**: Perform eigenvalue decomposition on the covariance matrix to obtain eigenvalues and corresponding eigenvectors.

**Selecting principal components**: Sort the feature vectors in descending order based on the size of their eigenvalues. The larger the eigenvalue, the greater the variance of the data in that direction and the more information it contains. Select the eigenvectors corresponding to the first few largest eigenvalues, which are the principal components.

**Constructing a projection matrix**: The selected feature vectors form a new spatial basis, forming the projection matrix. After multiplying the original dataset by this projection matrix, it is transformed into a new space, achieving dimensionality reduction. Finally, project the original dataset onto the selected principal components to obtain the reduced dimensional data. These data are presented in a new feature space and retain the most important statistical attributes.

In high-dimensional data, by selecting the first few principal components, the dimensionality of the data can be reduced while retaining most of the information. The selection of the number of principal components depends on the proportion of variance to be explained, usually determined by the variance explained rate. We have chosen a 90% explanatory power in our project, corresponding to the first 22 principal components. PCA can remove noise from data and improve data quality.

**4.Four different clustering methods**

**Partitioning Around Medoids clustering method**:

PAM is a distance based clustering algorithm that is particularly suitable for datasets with high feature dimensions, few data points, and sensitivity to outliers. The steps of PAM clustering method:

Initialization: Randomly select k data points as the initial medoids (center points).

Construction: For each point in the data set, calculate the distance between it and all medoids. The point is assigned to the nearest medoid, forming k clusters.

Exchange: For each cluster, consider replacing the current medoid with another point in that cluster. If the replacement can reduces the total distance of the cluster.

Iteration: Repeat steps 2 and 3 until there is no better medoid to replace, or a preset number of iterations is reached.

End: When the clustering no longer changes or reaches a certain number of iterations, the algorithm ends and outputs the final medoids and clustering results.

Gaussian Mixture Model (GMM):

The Gaussian Mixture Model (GMM) is a probabilistic clustering algorithm that is particularly suitable for datasets where the data points are assumed to be generated from multiple Gaussian distributions.

Initialization: Randomly select the initial parameters for k Gaussian distributions, including the mean vectors, covariance matrices, and mixing weights.

Expectation Step (E-step): For each data point, compute the probability that it belongs to each Gaussian component, i.e., the posterior probabilities.

Maximization Step (M-step): Based on the probabilities calculated in the E-step, re-estimate the parameters of each Gaussian component to maximize the likelihood function of the dataset.

Iteration: Repeat the E-step and M-step until the parameters converge or a predefined maximum number of iterations is reached.

Termination: When the parameter changes are no longer significant or the maximum number of iterations has been reached, the algorithm terminates and outputs the final Gaussian component parameters and clustering results.

**Hierarchical Clustering**:

Hierarchical clustering is a distance-based clustering algorithm that generates a dendrogram, which displays the hierarchical relationships among data points.

Initialization: Each data point is treated as a separate cluster.

Aggregation: Calculate the distances between all clusters and merge the two closest clusters to form a new cluster.

Iteration: Repeat the aggregation step until all data points belong to a single cluster or a predefined stopping condition is met.

Termination: When all data points have been merged into one cluster or the stopping condition is satisfied, the algorithm terminates and provides the final cluster structure.

**K-Means Clustering**:

K-Means is a distance-based clustering algorithm that is suitable for datasets where the data points are distributed in spherical clusters.

Initialization: Randomly select k data points as the initial cluster centers.

Assignment: For each data point, calculate its distance to all cluster centers and assign it to the nearest cluster center.

Update: Recalculate the new center of each cluster, typically as the mean of all points in the cluster.

Iteration: Repeat the assignment and update steps until the cluster centers no longer change significantly or a predefined maximum number of iterations is reached.

Termination: When the cluster centers no longer change significantly or the maximum number of iterations is reached, the algorithm terminates and outputs the final cluster centers and clustering results.

**5.Machine Learning Details**

In our study, we used machine-learning classifiers for identify high-risk PD patients. This classifiers were implemented using R package caret [1], which provides a nice interface to access many machine-learning algorithms in R. Furthermore, it also provides a user-friendly framework for training different machine-learning models. We used parameter configurations of machine learning that were previously defined by Fernandez-Delgado et al [2], and we have listed the classification methods along with their parameters and corresponding R packages. At last, we focused on and validated the Decision tree model because of its better performance and ease-to-use.

**Decision tree (DT)**

A C5.0 decision tree based classification method was used in the analysis. C5.0 function of the “C50” package was used for creating classification trees with default parameter tuning under caret interface. The hyperparameter selection and cross validation of decision trees can be seen in Figure S2.

**
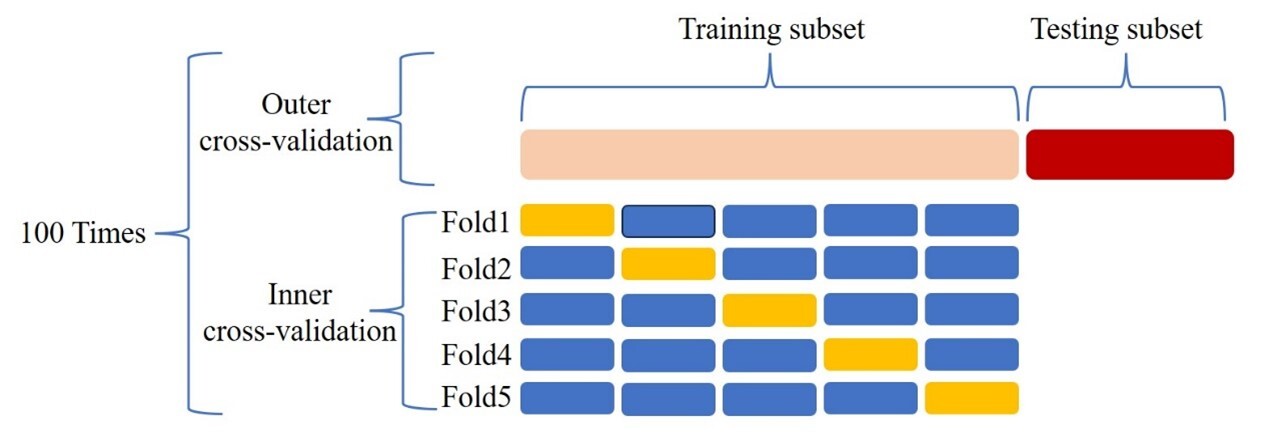
**

**Figure S2.** The cross validation process of decision tree (DT) algorithm based on training queue

**Table S3. Multifactor logistic regression analysis based on the entire PPMI dataset**

| Variable |  | | | |  |
| --- | --- | --- | --- | --- | --- |
|  | Univariate analysis |  | Multivariate analysis |  |  |
|  | OR | P value | OR | P value |  |
| Age | 1.01 (0.969 1.052) | 0.647 | NA | NA |  |
| Gender | 1.155 (0.588, 2.268) | 0.675 | NA | NA |  |
| EDUCYRS | | 0.912(0.832, 1.000) | 0.049* | NA | NA |
| Family genetic history | 1.375 (0.688, 2.747) | 0.367 | NA | NA |  |
| Hoehn–Yahr stages | 0.607(0.306, 1.202) | 0.152 | NA | NA |  |
| PD subtype | 1.536(1.029, 2.292) | 0.036* | 1.542(1.07, 2.223) | 0.02* |  |
| UPDRS1 | 1.113 (1.029, 1.204) | 0.008* | 1.542(1.07, 2.223) | 0.02* |  |
| UPDRS2 | 1.045 (0.982, 1.112) | 0.165 | NA | NA |  |
| UPDRS3 | 1.011 (0.965, 1.06) | 0.632 | NA | NA |  |
| ESS | 0.762 (0.289, 2.008) | 0.582 | NA | NA |  |
| RBD | 1.839 (1.081, 3.128) | 0.025 | NA | NA |  |
| abeta | 0.999(0.998, 1.000) | 0.057 | NA | NA |  |
| asyn | 1.000 (0.999, 1.001) | 0.631 | NA | NA |  |
| tau | 1.006 (1.001, 1.010) | 0.017* | NA | NA |  |
| ptau | 0.969 (0.743 1.264) | 0.018* | 1.006(1.001, 1.011) | 0.014* |  |
| MCI | 2.992(1.517, 5.903) | 0.002* | NA | NA |  |
| GM volume | 2.068(0.516, 8.296) | 0.01* | 3.379(1.646, 6.934) | 0.001* |  |
| WM volume | 3.246(1.219, 8.641) | 0.038 | NA | NA |  |
| CSF volume | 3.657 (1.287, 10.392) | 0.015 | NA | NA |  |

Note: NA, not available because the variable is not included in multiple variables. The P value indicates whether the variable is an independent predictor of PD-MCI. *, P<0.05. EDUCYRS: Categorical Education; H-Y stages: Hoehn-Yahr stages; Ess: Epworth Sleepiness Scale; RBD: rapid-eye-movement behavior disorde; MCI: Mild cognitive impairment; UPDRS: Unified Parkinson's Disease Rating Scale; GM: Gray Matter; WM: White Matter; CSF: Cerebrospinal Fluid.

**References**

1. Kuhn M. Building predictive models in R using the caret package. J Stat Softw. 2008, 28(5):1–26.
2. Fernández-Delgado, Cernadas, Barro, et al. Do we need hundreds of classifiers to solve real world classification problems? J. Mach. Learn. Res. 2014, 15:3133–3181.
